# Muscle contractions and pain sensation accompanying high-frequency electroporation pulses

**DOI:** 10.1038/s41598-022-12112-9

**Published:** 2022-05-16

**Authors:** Aleksandra Cvetkoska, Alenka Maček-Lebar, Peter Trdina, Damijan Miklavčič, Matej Reberšek

**Affiliations:** 1 Faculty of Electrical Engineering, University of Ljubljana, Tržaška 25, 1000 Ljubljana, Slovenia; 2 Institute of Clinical Neurophysiology, University Medical Centre Ljubljana, Zaloška 2, 1000 Ljubljana, Slovenia

**Keywords:** Biological techniques, Neuroscience

## Abstract

To minimize neuromuscular electrical stimulation during electroporation-based treatments, the replacement of long monophasic pulses with bursts of biphasic high-frequency pulses in the range of microseconds was suggested in order to reduce muscle contraction and pain sensation due to pulse application. This treatment modality appeared under the term high-frequency electroporation (HF-EP), which can be potentially used for some clinical applications of electroporation such as electrochemotherapy, gene electrotransfer, and tissue ablation. In cardiac tissue ablation, which utilizes irreversible electroporation, the treatment is being established as Pulsed Field Ablation. While the reduction of muscle contractions was confirmed in multiple in vivo studies, the reduction of pain sensation in humans was not confirmed yet, nor was the relationship between muscle contraction and pain sensation investigated. This is the first study in humans examining pain sensation using biphasic high-frequency electroporation pulses. Twenty-five healthy individuals were subjected to electrical stimulation of the tibialis anterior muscle with biphasic high-frequency pulses in the range of few microseconds and both, symmetric and asymmetric interphase and interpulse delays. Our results confirm that biphasic high-frequency pulses with a pulse width of 1 or 2 µs reduce muscle contraction and pain sensation as opposed to currently used longer monophasic pulses. In addition, interphase and interpulse delays play a significant role in reducing the muscle contraction and/or pain sensation. The study shows that the range of the optimal pulse parameters may be increased depending on the prerequisites of the therapy. However, further evaluation of the biphasic pulse protocols presented herein is necessary to confirm the efficiency of the newly proposed HF-EP.

## Introduction

Electroporation/electropermeabilization describes the phenomenon where the cell membrane is exposed to sufficiently strong electric field that is generated by short-duration, high-voltage pulses. This induces a transmembrane voltage (TMV), e.g., 500 mV, which far exceeds its resting TMV (typically -40 mV to -70 mV). Thus, plasma membrane permeability is increased and transmembrane transport of molecules is enabled which otherwise are unable to cross the membrane^1^. Electroporation can be either reversible, when the cell recovers after the treatment and survives, or irreversible when the exposure leads to cell death^2–4^.

Electroporation is used in multiple clinical applications^5–7^ as well as in biotechnology^8^, food processing^9^, and environmentally relevant applications^10^. Reversible electroporation is successfully used as combination of high-voltage pulsed electric fields with low-permeant chemotherapeutic drug or with DNA: electrochemotherapy (ECT) and gene electrotransfer (GET), respectively^11–17^. On the other hand, irreversible electroporation (IRE) appeared as a new medical application^3^ for non-thermal tumor^18–20^ and cardiac ablation (Pulse Field Ablation–PFA)^21–23^ providing considerable benefits over existing thermal ablation methods, such as reducing the risk of damaging the nearby critical tissue. Especially in cardiac electrophysiology, PFA may represent a dominant future treatment^24–26^.

Currently, in most of the electroporation-based clinical applications, relatively long monophasic pulses of 50–100 μs are delivered with low repetition rates, synchronized with the heart rhythm^3,27,28^. In gene therapy vaccination even longer pulses (in the range of milliseconds) e.g., 50 ms are applied^17^. The electric field thresholds required to initiate electroporation are higher than the thresholds that trigger action potentials in excitable cells, which means that electroporation is not successfully achieved without (unintended) electrical stimulation of excitable cells^29,30^, i.e., muscle and nerve cells. Consequently, delivery of electroporation pulses leads to muscle contraction and sensory nerve cells (e.g., nociceptors) excitation rendering these treatments unpleasant or even painful. Muscle contraction may potentially lead to interference with the heart rhythm and/or displacement of the electrodes during the treatment, which increases the complexity of the treatments and may pose a risk to the patient. The patients need to undergo local or general anesthesia, receive muscle relaxants to ensure adequate neuromuscular blockade and proper respiratory function^31–34^, and pulse delivery needs to be synchronized with the patient´s ECG^27^.

Stimulation of nerves and muscles has been extensively investigated in the past, showing that short pulses and higher frequencies of alternating current (up to 10 kHz) can increase sensory, motor, and pain thresholds^35–39^. Thus, to minimize stimulation of muscle and nerves during electroporation-based treatments, the increase of the pulse repetition frequency far above the frequency of tetanic contraction was suggested. This was confirmed to be an effective treatment showing reduction of the overall muscle contractions and pain sensation^40,41^. Recently, a new waveform was suggested that could potentially replace the standard 50–100 μs monophasic pulses: a burst of short biphasic pulses (with pulse width from 1 to 10 μs) with the same total ‘on-time’ of the pulses (energized time)^42–44^ and with pulse repetition rates ranging from 250 kHz to 1 MHz. It was shown that such pulses could be used for tissue ablation while potentially avoiding muscle contractions. This novel electroporation waveform appeared under the term high-frequency irreversible electroporation (H-FIRE)^43,44^. Moreover, a numerical modeling study^45^ also suggested that by using bursts of short biphasic pulses, the same IRE efficiency for tissue ablation can be achieved while avoiding action potential triggering in the nerve fibers nearby that would be stimulated by the use of long monophasic pulses. The encouraging results obtained from the initial studies led to a series of experiments to study H-FIRE^42,46–54^. Additionally, it was demonstrated that high-frequency (HF) pulses can be efficiently used to ablate tumors^47^, cardiac tissue^23,26,55^ and potentially also for ECT^56^ and GET^57^. However, the data obtained through cell/animal experiments, modeling, and theoretical considerations although of great value, do not allow to evaluate pain reduction during HF electroporation therapy. Moreover, the correlation between muscle contraction and pain sensation during the pulse treatment has not been examined yet. There can be differences in excitation, as the signals are transmitted via different fibers—myelinated (A-alpha, beta, gamma, delta) or unmyelinated (C-fibers), with A-delta and C-fibers being the major pain-conducting nerve fibers^58,59^.

In our study, we examined pain sensation during the pulse treatment and the correlation between the elicited muscle contraction and pain sensation caused by short biphasic HF pulses in healthy individuals. Additionally, we investigated the relationship between muscle contraction and pain sensation while varying the pulse parameters (pulse width, interphase and interpulse delays). Finally, we analyzed which pain fibers have the higher possibility of being excited (A-delta or C-fibers) based on the pain descriptors selected from the pain questionnaires by the individuals.

## Materials and methods

**Figure 1:**
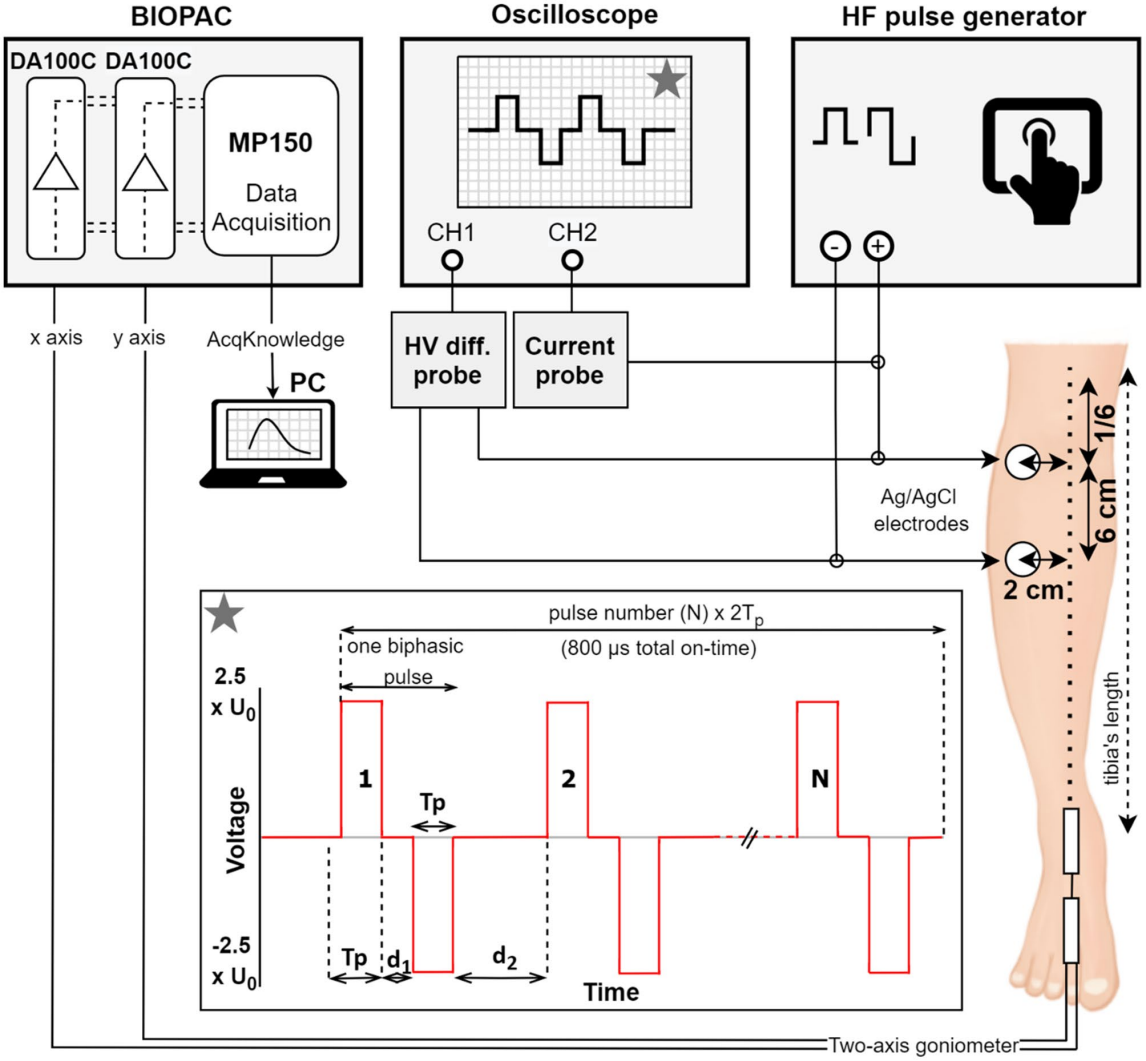
Experimental setup and electrodes/goniometer placement. Stimulation pulses were delivered via electrodes connected to the HF pulse generator. The electrodes (marked with circles) were placed on the right leg: the upper electrode was placed on 1/6th of the tibia’s length, the lower electrode was placed 6 cm lower. Both electrodes were placed 2 cm right lateral to the bone (left in the figure). The output pulses were monitored on an oscilloscope using high-voltage (HV) differential and current probe. Asterisk: applied pulses—biphasic pulses with 800 μs total on-time. T_p_-pulse width (equal for positive and negative phase), d_1_-interphase delay, d_2_-interpulse delay, N-number of pulses. The response from the ankle (muscle contraction) was acquired with twin-axis goniometer connected to the Biopac unit. The data was analyzed on a personal computer (PC) using the AcqKnowledge software. DA100C-amplifier, MP150-data acquisition system.

In our study, 25 healthy individuals participated. Muscle contraction was initiated by electrical stimulation of the tibialis anterior muscle on the right leg. As this muscle acts primarily in ankle dorsiflexion, the angle of ankle dorsiflexion was measured by a two-axis goniometer. Due to different insulating properties of the skin and subcutaneous tissue of the individuals, strength-duration curves were determined for monophasic and biphasic pulses with different pulse widths for each individual. Based on the amplitude of 8 monophasic pulses with a pulse width of 100 μs, delivered with 5 kHz pulse repetition rate, which results in measurable muscle contraction, the stimulus amplitude for biphasic pulse protocols was determined. Biphasic pulses with pulse width from 1 to 5 μs were tested while changing the interphase delay (time between positive and negative phase) and interpulse delay (time between the pulses) (Fig. 1). Each individual was subjected to a randomly selected group of 30 biphasic pulse protocols for muscle contraction determination. In order to examine the pain sensation and assess pain intensity and unpleasantness, each individual was requested to fill short-form McGill pain questionnaires for randomly selected half of the delivered biphasic pulse protocols after the stimulation.

### Participants

The research was approved by the National Medical Ethics Committee of Slovenia (Doc. no. 0120–61/2020) and was conducted following the Declaration of Helsinki, Convention on Human Rights and Biomedicine (ETS No.164), and the Slovenian Code of Medical Ethics. Informed consent was obtained from all individuals before the start of the measurements. All of them were given the opportunity to withdraw from the study at any time, even after signing the informed consent to participate. Thirty healthy individuals volunteered to participate in the study. Five of the individuals were not included in the study due to too strong muscle contraction when the muscle was stimulated with the lowest possible amplitude of the pulse generator. The main set of measurements was thus performed on 25 individuals (12 females and 13 males) in the age range from 22 to 58 years (mean: 32.5 years, median: 27 years). Twenty individuals were younger (range: 20–32 years) and 5 elder (range: 52–58 years).

### Experimental setup

For the delivery of electrical pulses, a prototype high-frequency (HF) pulse generator was used (University of Ljubljana, mPOR, Slovenia). Before measurements, the electrical safety of the pulse generator and measuring system was verified with a certified and calibrated electrical safety analyzer Fluke ESA620 (Fluke Biomedical, Washington, USA) for medical devices following the medical standard IEC 60,601–1. The available energy of the pulse generator was physically limited to 5 J with the capacitance of the integrated supply capacitor, enabling safe delivery of pulses and preventing potential damage to the skin. The lowest amplitude limit of the pulse generator was 60 V; the highest amplitude limit was 1000 V. The pulses delivered were monitored by a high-voltage (HV) differential probe HVD3605A (LeCroy, USA), current probe CP031 (LeCroy, USA) and HDO6000 High-Definition oscilloscope (LeCroy, USA) via power medical isolation transformer.

Measurements were performed on the right leg in all individuals. Self-adhesive Ag/AgCl electrodes (3 M™ Red Dot™, 3 M, Minnesota, USA) for single use were connected to the pulse generator via lead wires with a clip. Before measurement, the skin was cleaned with 70% ethanol and the participant’s tibia length was determined^60^ in order to place the electrodes consistently for each individual. The upper electrode was placed on 1/6th of the tibia’s length and the lower electrode was placed 6 cm lower. Both electrodes were placed 2 cm right, lateral to the bone (Fig. 1).

To determine muscle contraction, the angle of ankle dorsiflexion was measured with twin-axis goniometer TSD120B (Biopac Systems, Inc., USA). The upper mounting point was placed on the lower part of the right tibia and the lower mounting point was placed on the right forefoot, above the extensor tendons (Fig. 1). The goniometer was attached using double-sided tape and was additionally secured with single-sided tape. Two planes of angular movement were simultaneously measured (foot dorsiflexion/plantarflexion and abduction/adduction). Each channel of the goniometer was connected to an DA100C amplifier as part of the MP150 data Acquisition system (Biopac Systems Inc., USA). The AcqKnowledge 4.1 software (Biopac Systems Inc., USA) was used for real-time measurements and recording of the signals (muscle contraction responses) as MATLAB data files for further analysis in MATLAB vR2018a (MathWorks Inc., Natick, MA, USA). Calibration of the goniometer was performed before each measurement using the software calibration features. The complete experimental setup is shown in Fig. 1.

In preliminary measurements, we examined how the position of the electrodes affected the muscle contraction as measured by ankle dorsiflexion. The results showed that moving the electrodes for 1–2 cm proximally/distally does not considerably affect the results while moving the electrodes for more than 4 cm laterally from the bone requires higher amplitudes (more than 20%) to be delivered to achieve the same muscle contraction response (data not shown).

### Test procedure

The measurements were performed in the Laboratory for Physiological Measurements (Faculty of Electrical Engineering, University of Ljubljana, Slovenia). The duration of the measurements was approximately one hour and thirty minutes per individual. No anesthetics or nerve blockers were used during the measurements. Before the measurements, the protocol was explained to each individual. There were no serious side effects recorded, nevertheless a medical doctor was always available during the measurements. The only side effect noticed during or after measurements was slight redness at the site of the electrodes after the treatment, which disappeared within few hours. None of the individuals withdrew from the study due to pain or other reasons although they had the opportunity to withdraw from the study at any time.

#### Strength–duration curves

In the first part (approximately 30 min) of the measurements, we determined two strength-duration (S-D) curves per individual. These curves represent the stimulus strength (voltage) needed to produce minimal muscle contraction for certain pulse width and pulse type (monophasic/biphasic)^58,61–63^. Thus, for each individual, stimulation for one monophasic and one biphasic pulse for five pulse widths (T_p_: 1, 2.5, 5, 10, or 50 µs) was performed. For the biphasic pulses, the interphase delay (delay between positive and negative phase) was randomly chosen (d_1_: 1, 2.5, 5, 10, or 100 μs). Each S-D curve was measured by first applying the longest pulse width (50 µs) to the muscle and increasing the stimulus intensity (amplitude) until a lower limit of quantification (LLOQ) of muscle contraction (muscle response) was reached, defined as an angle of 3.6° to 4° ankle dorsiflexion. The LLOQ is the lowest angle that can be quantitatively determined with suitable precision and accuracy by our measurement system. Subsequently, the pulse width was decreased to 10 µs and the amplitude increased until the LLOQ of muscle contraction response was obtained. This process was repeated for pulse widths of 5, 2.5, and 1 µs. Thus, five points for each S-D curve were determined. Note that monophasic pulses were 1xT_p_ long while biphasic pulses were 2xT_p_ long; e.g., monophasic: 50 µs; biphasic: 50 µs positive + 50 µs negative = 100 µs.

#### Determining the stimulus amplitude for the measurements

In the same way as was the amplitude for one point on the S-D curve determined, the stimulus amplitude for 8 monophasic pulses with a pulse width of 100 μs, delivered with 5 kHz pulse repetition rate (5 pulses per one millisecond) was determined. This pulse protocol was chosen to be the amplitude determining (reference pulse protocol), as it is the most often used electroporation protocol^64^ in clinical practice. The pulses were delivered with an initial amplitude of 60 V, gradually increased in small increments (5–10 V) until minimal muscle contraction was obtained. As the pulse generator was not able to deliver pulses of amplitudes lower than 60 V, five of the individuals who initially volunteered for the study were not included, due to too strong muscle contraction, i.e., above 4.6° ankle dorsiflexion when the muscle was stimulated with an amplitude of 60 V.

#### Biphasic pulse protocols

Twenty-five sets of biphasic pulse protocols were generated and coded with numbers from 1 to 25 at the beginning of the study, each set containing 30 randomly chosen biphasic pulse protocol numbers, within which 15 were randomly chosen for the pain questionnaires (www.random.org, RANDOM, Ireland). All of the biphasic pulse protocols were repeated nearly equal times. Before measurements, each individual drew a set number (1—25) of biphasic pulse protocols. Thus, each individual received 30 biphasic pulse protocols in addition to the reference protocol and filled out 15 pain questionnaires. There was a 2 min waiting time between each protocol. This second part of the measurements took approximately one hour.

All biphasic pulse protocols used in the study had the same total on-time as the reference pulse protocol (8 × 100 µs, 5 kHz). Therefore, in the biphasic pulse protocols the number of pulses and pulse width (duration of each phase) were changed so that the total on-time of the pulses (N x 2T_p_) was the same, i.e., 800 µs as shown in Fig. 1 (insert marked with asterisk). Additionally, for each pulse width tested (1 µs to 5 µs), the interphase d_1_ (time between the end of the positive and beginning of the negative phase of the pulse) and interpulse delay d_2_ (time between the end of the negative pulse and beginning of the new positive pulse) were changed. The interpulse delay d_2_ was equal to or longer than the interphase delay (d_2_ ≥ d_1_) in each pulse protocol. The pulse repetition rate (PRR) was calculated as PRR = 1 / (2T_p_ + d_1_ + d_2_). The total number of biphasic pulse protocols examined was 51 (see Table S1 in the Supplementary files). The amplitude used for the biphasic pulse protocols was 2.5 times higher than the amplitude determined for the reference pulse protocol, since higher amplitudes are required for biphasic pulses to obtain comparable effect as with monophasic pulses^42,56,65^ at the same total on-time. For example, if the amplitude determined for 8 monophasic pulses was U_0_ = 100 V, the amplitude for the biphasic pulse protocols was 100 V x 2.5 = 250 V. Table 1 shows the values of all pulse parameters used in the study.

**Table 1:** Values of the pulse parameters for all pulse protocols included in the study.

Polarity	Pulse width (T_p_) [µs]	N	d_1_ [µs]	d_2_ [µs] (d_2_ ≥ d_1_)	Amplitude (U)
Monophasic	100	8	/	100 (5 kHz)	U_0_
Biphasic	1	400	1, 2, 5, 10, 100	1, 2, 5, 10, 100	2.5 × U_0_
Biphasic	2	200	1, 2, 5, 10, 100	1, 2, 5, 10, 100	2.5 × U_0_
Biphasic	3	133	1, 5	1, 5, 800	2.5 × U_0_
Biphasic	4	100	1, 5	1, 5, 800	2.5 × U_0_
Biphasic	5	80	1, 2, 5, 10, 100	1, 2, 5, 10, 100	2.5 × U_0_

All biphasic pulse protocols have equal total on-time, i.e., N × 2Tp = 800 µs.

Tp pulse width (equal for positive and negative phase); N number of pulses; d1 interphase delay; d2 interpulse delay.

#### Pain questionnaires

All individuals completed a short-form McGill Pain Questionnaire (SF-MPQ)^66^ for 15 randomly chosen biphasic pulse protocols (out of the 30 examined biphasic pulse protocols for muscle contraction responses) immediately after the delivery of the specific biphasic pulse protocol, to examine the nature of pain and assess pain intensity and unpleasantness. Twenty-two individuals completed the Slovenian version, and three individuals completed the English version of the SF-MPQ, as Slovenian was not their native language. Every pain questionnaire consisted of four parts. The first part was the main component—Pain Rating Index (PRI) of the SF-MPQ, which was used to determine the sensory (pain descriptors 1–11) and affective (pain descriptors 12–15) components of pain, rated on an intensity scale as 0 = none, 1 = mild, 2 = moderate or 3 = severe. The second part of the SF-MPQ referred to two separate 10 cm horizontal Visual Analog Scales (VAS)^67^ which were used to assess pain intensity and unpleasantness, respectively. Both were marked as “no pain/no unpleasantness” on one end and “most intense pain/worst possible unpleasantness” on the other end. In the third part, the SF-MPQ included the Present Pain Intensity (PPI) index, which was a five-point interval scale measuring the intensity of overall pain. The scale ranged from “mild” to “excruciating” with points from 0 to 5, respectively, and evaluated the intensity of overall pain experienced during electrical pulse delivery for each specific biphasic pulse protocol. The fourth, i.e., the last part was referred to answering three questions about the exact position of pain in the body, the duration of the pain sensation, and the pain sensation changing with time. After taking off the electrodes, each individual answered two questions regarding the sensitivity of the skin and visible signs of skin injury immediately and 6 h after the end of the measurements.

##### Calculation of the total pain index

The total pain index was calculated as a sum of the Pain Rating Index (PRI) and both visual analogue scales (VAS). PRI was derived from the sum of the intensity rank values of the words chosen by each individual for sensory and affective descriptors (15 pain descriptors, scale: 0–3). VAS analysis consisted of measuring the distance in centimeters by a ruler between the start of the line on the left side and the mark made by the individual (scale: 0–10). Therefore, the maximum value of the pain index from the pain questionnaires was 65 (15 × 3 + 2 × 10 = 65). The Present Pain Intensity (PPI, scale: 0–5) was not included in the calculation of the total pain index, however, it was used to estimate the overall pain intensity.

##### Pain descriptors

Descriptors included in the first part of the pain questionnaire were analyzed to determine which pain fibers have the higher possibility of being excited (A-delta or C-fibers). According to the literature, A-delta fibers mediate rapid nociception or first pain, typically characterized as sharp, pricking pain, while C-fibers mediate dull, aching pain, which can often be difficult to localize^68–70^. Thus, three pain descriptors for each type of fiber were chosen from the pain questionnaire: shooting, stabbing, and sharp as representative of A-delta fibers and throbbing, cramping, and aching as representative of C-fibers. Based on the intensity of the chosen descriptor from each individual, a mean intensity for each descriptor was calculated separately for each pulse protocol.

### Statistical analysis

Thirty different biphasic pulse protocols per individual were delivered in order to obtain 30 muscle contraction responses (angles of ankle dorsiflexion) and 15 pain indexes, as the pain questionnaires were filled only for half of the delivered biphasic pulse protocols. The mean and median values were calculated separately for the muscle contraction responses and pain indexes for each pulse protocol. Collected data showed non-normal distribution as tested with the Sharpio-Wilk’s test. Therefore, the data were transformed using inversed square root (for muscle contraction responses) and square root (for pain indexes). Both transformations were performed in Design Expert v.12 (Stat-Ease, Inc., Minneapolis, USA) and resulted in a normal distribution. To compare the transformed data with the biphasic pulse protocols^71^, an N-way repeated measures analysis of variance (rmANOVA) was used. Multiple comparison test for three factors (pulse width, interphase, and interpulse delay) using the Dunn and Sidak’s approach^71^ was performed in order to compare intervals among the pulse protocols and find statistically different pulse protocols (separately for muscle contraction responses and pain indexes). The statistical analysis was performed in MATLAB vR2018a (MathWorks Inc., Natick, MA, USA). For all tests, the level of significance was set to 0.05.

### Clustering

As there were 51 biphasic pulse protocols, clustering (of protocols) was performed using a hierarchical cluster tree (dendrogram) in MATLAB vR2018a (MathWorks Inc., Natick, MA, USA). An average was calculated from the transformed data described in the previous subsection. The average values were then inversely transformed and normalized. Thus, each pulse protocol was represented by a pair of two coordinates (x-muscle contraction response; y-pain index). The distance between each pair of pulse protocols was calculated using the Euclidean distance and based on the average distances, two points were linked together. According to the dendrogram, five clusters were generated. Each cluster consisted of the biphasic pulse protocols that were close to each other.

### Additional measurements

#### Biphasic pulse protocols with extended interpulse delay (d_2_)

Based on a recent theoretical/numerical study^72^, we also investigated if extending the interpulse delay (d_2_) beyond 10–100 µs reduces the muscle contraction response and increases the pain. These additional measurements were performed on 10 individuals that were included in the first part of the study and volunteered again. Sixteen additional biphasic pulse protocols were derived with extended d_2_ (200, 500, 750, and 1000 µs) and the interphase delay (d_1_) and pulse width set to either 1 or 5 µs (Table 2). Thus, the pulse repetition rates were ranging from approximately 5 kHz (for d_2_ = 200 µs) to 1 kHz (for d_2_ = 1000 µs). The amplitude was determined in the same way as described previously (in the subsection: ‘’Determining the stimulus amplitude for the measurements’’). All 16 new biphasic pulse protocols were delivered on each individual in a random order. For comparison with previously used shorter interpulse delays, the pulse protocols with d_2_ = 10 and 100 µs were also delivered. For each pulse protocol delivered, the individuals were requested to fill the short-form McGill pain questionnaire.

**Table 2:** Additional biphasic pulse protocols delivered.

Polarity	Pulse width (T_p_) [µs]	N	d_1_ [µs]	d_2_ [µs]	Amplitude (U)
Biphasic	1	400	1, 5	200, 500,750,1000	2.5 × U_0_
Biphasic	5	80	1, 5	200, 500,750,1000	2.5 × U_0_

All biphasic pulse protocols have equal total on-time, i.e., N × 2Tp = 800 µs.

Tp pulse width; N number of pulses; d1 interphase delay; d2 interpulse delay.

#### Interchanged interphase (d_1_) and interpulse delays (d_2_)

The biphasic pulse protocols tested in the study were chosen so that the interpulse delay (d_2_) was always equal to or longer than the interphase delay (d_1_), d_2_ ≥ d_1_. Additional measurements were performed on 10 individuals that were included in the first part of the study and asked to volunteer again in order to investigate the muscle contraction response and pain index when d_1_ was longer than d_2_ (interchanged delays, d_1_ > d_2_). Six of the 51 biphasic pulse protocols previously tested were chosen which had the highest difference between the values of d_1 _and d_2_, as for these delays the highest deviations in the results were expected. Three pulse protocols were chosen in order to test the effect of the pulse width (T_p_ = 1, 2, and 5 µs) when d_1_ and d_2_ were interchanged. The other three pulse protocols were with different delays (highest possible difference between d_1 _and d_2_), but all with a pulse width of 5 µs. Therefore, six additional biphasic pulse protocols with interchanged delays were generated. For comparison, the six old protocols (before the interchange) were also delivered in random order. For each pulse protocol delivered, the individuals were requested to fill the short-form McGill pain questionnaire.

## Results

### Strength-Duration curves

**Figure 2:**
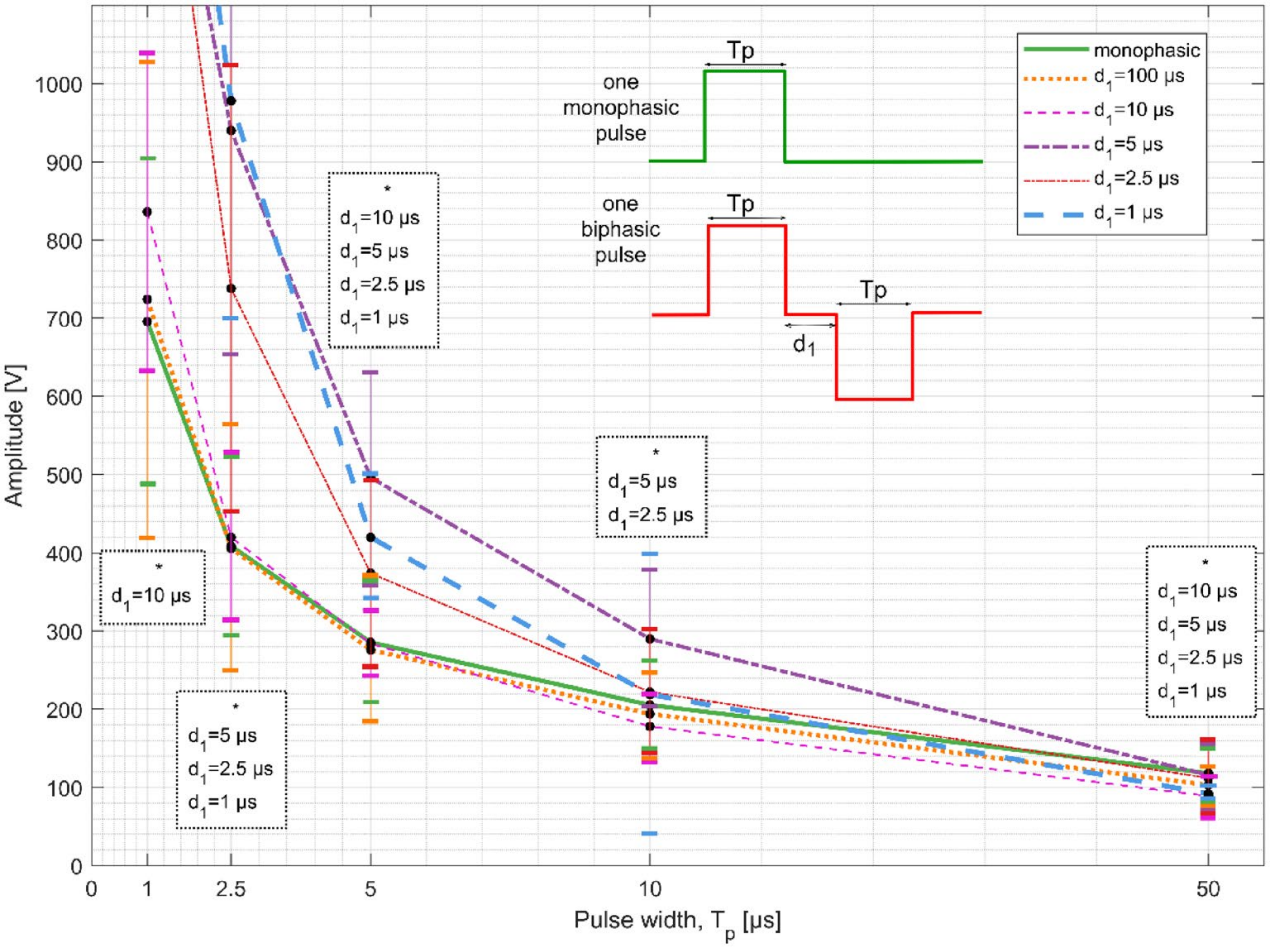
Threshold amplitude as a function of the pulse width for single monophasic (solid green curve) and biphasic pulses (Strength–Duration curves). Biphasic pulses are shown for each interphase delay from 1 µs to 100 µs. The results are shown as mean amplitude of the individuals (black dots) ± standard error (vertical bars). The boxes with asterisks (*) and interphase delays show statistically significant differences between the monophasic pulse and marked interphase delay (biphasic pulse) for each pulse width tested (statistically higher mean values for the biphasic pulses for all pulse widths tested except for T_p_ = 50 µs). Note that for pulse width of 1 µs, paired t-test was performed only for d_1_ = 10 µs and 100 µs, as the threshold amplitude was higher than 1000 V for the rest of the interphase delays.

In Fig. 2, we present the mean strength-duration (S-D) curves obtained for single monophasic (each dot is a mean value of 25 measurements in 25 individuals) and biphasic pulse with various interphase delays (each dot is a mean of five measurements in five individuals). Measurements were performed at five pulse widths T_p_ = 1, 2.5, 5, 10, and 50 µs. For each pulse width, the threshold amplitude (pulse amplitude where minimal muscle contraction was observed) was recorded as a point on the graph. The thresholds rise as the pulse width and interphase delay (delay between the positive and negative pulse) are shortened. Biphasic pulses with short interphase delays (d_1_ = 1, 2.5, and 5 µs) have higher threshold amplitudes than monophasic pulses or biphasic pulses with longer delays (d_1_ = 10 and 100 µs) for T_p_ = 1, 2.5, 5, and 10 µs. Biphasic pulse (for all interphase delays) and monophasic pulse at all pulse widths were compared using paired t-test (with a level of significance set to 0.05). As expected for T_p_ = 50 µs, there was statistically significant difference between single monophasic pulse and biphasic pulse with d_1_ = 1, 2.5, 5, and 10 µs (statistically lower mean values for the biphasic pulses) as for biphasic pulse the total duration was 2xT_p_ and for monophasic 1xT_p_ (Fig. 2). For all other tested pulse widths, there was a statistically significant difference between single monophasic pulse and biphasic pulse (but the mean values for the biphasic pulses were statistically higher) for the interphase delays stated in the boxes and marked with asterisks on Fig. 2. For a pulse width of 1 µs paired t-test was performed only for d_1_ = 10 µs and 100 µs, as the threshold amplitude was higher than 1000 V (highest possible amplitude the pulse generator was able to deliver) for the rest of the interphase delays.

### Clustering

**Figure 3:**
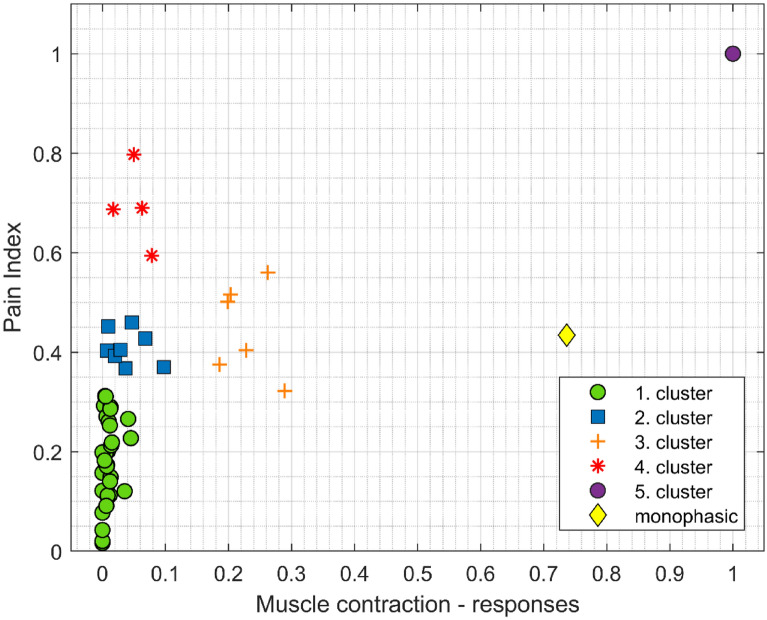
Clustering based on a hierarchical cluster tree (dendrogram). Each mark represents one pulse protocol: x—muscle contraction response, y—pain index. The data shown is normalized based on the purple cluster (T_p_ = 5 µs, d_1_ = 100 µs, d_2_ = 100 µs). Note that the yellow diamond represents the amplitude determining (reference) protocol (8 monophasic pulses × 100 µs, 5 kHz) with 2.5 lower amplitude.

Using the hierarchical cluster tree (dendrogram) with normalized data, five clusters based on similar/different muscle contraction responses and pain sensation were identified. The hierarchical cluster tree (Fig. S1) along with the table of biphasic pulse protocols and suitable coloring (Table S1) can be found in the Supplementary files. In Fig. 3, we present all 51 biphasic pulse protocols marked and colored according to the cluster they belong to. Each symbol represents the average of one pulse protocol: x-muscle contraction response, y-pain index in the coordinate system. The data is normalized based on the single-cluster pulse protocol with parameter values: T_p_ = 5 µs, d_1_ = 100 µs, d_2_ = 100 µs, which resulted in the highest muscle contraction response (6.2° ankle dorsiflexion) and highest pain index (13 out of 65). This is shown with a purple dot on the graph, i.e., coordinates (1, 1). Additionally, the amplitude determining (reference) protocol (8 monophasic pulses × 100 µs, 5 kHz) is marked with a yellow diamond. It is important to note that the amplitude for the reference protocol was always 2.5 times lower than the amplitude used for the biphasic pulse protocols, and the muscle contraction response was almost equal for each individual (minimal muscle response: 3.6°–4° of ankle dorsiflexion).

Four other clusters (green, blue, orange, and red) can be distinguished on the graph (Fig. 3). The green cluster (marked with green circles) barely causes any muscle contraction and has low pain index. In this cluster are mainly the pulse protocols that have short pulse width, T_p_ = 1 µs and 2 µs (Table S1 in the Supplementary files). The blue cluster (marked with blue squares) has almost similar muscle contraction responses, but slightly higher pain indexes than the green one. The pulse protocols in this cluster have very short interphase delays (d_1_) but longer pulse width (T_p_) and interpulse delays (d_2_) than the pulse protocols in the green cluster. The orange cluster (marked with orange crosses) has considerably higher muscle contraction response than the blue one at almost equal pain index. The pulse protocols that cause the highest muscle contraction response (orange cluster) all have T_p_ of 5 µs and d_1_ and d_2_ up to 10 µs. All biphasic pulse protocols except the single-cluster pulse protocol: T_p_ = 5 µs, d_1_ = 100 µs, d_2_ = 100 µs had lower muscle contraction responses than the reference protocol (marked with yellow diamond), i.e., 8 monophasic pulses × 100 µs, 5 kHz. When extending the interpulse delay above 10 µs, e.g., 100 µs, the muscle contraction response is reduced (pulse protocols marked with red asterisks) however, the pain index is increased. This indicates that the pain index does not necessarily correspond to the muscle contraction response and vice versa. In the clusters presented, the orange cluster is representative for higher muscle contraction response and the red cluster for higher pain index.

### Statistical analysis

In order to find statistically significant differences among the biphasic pulse protocols and support their clustering into “biphasic pulse protocols with higher muscle contraction response” and “biphasic pulse protocols with higher pain index”, an N-way repeated measures analysis of variance (rmANOVA) was performed on transformed data separately for muscle contraction responses and pain indexes. The complete graphs (Figs. S2 and S4) and tables (Figs. S3 and S5) are provided in the Supplementary files.

The pulse protocols marked with orange crosses in Fig. 3 are significantly different (higher means) from the pulse protocols with almost no muscle contraction response (green and blue cluster) when performing rmANOVA on the data for muscle contraction response. The pulse protocols marked with red asterisks in Fig. 3 are significantly different (higher means) from the pulse protocols with low pain index (green cluster) when performing rmANOVA on the data for pain indexes.

However, it is worth mentioning that when performing statistical analysis on the data for pain indexes, the statistical significance and clustering is different when the Pain Rating Indexes (PRI) and Visual Analogue Scales (VAS) are analyzed separately (data not shown).

### Biphasic pulse protocols with extended interpulse delay (d_2_)

**Figure 4:**
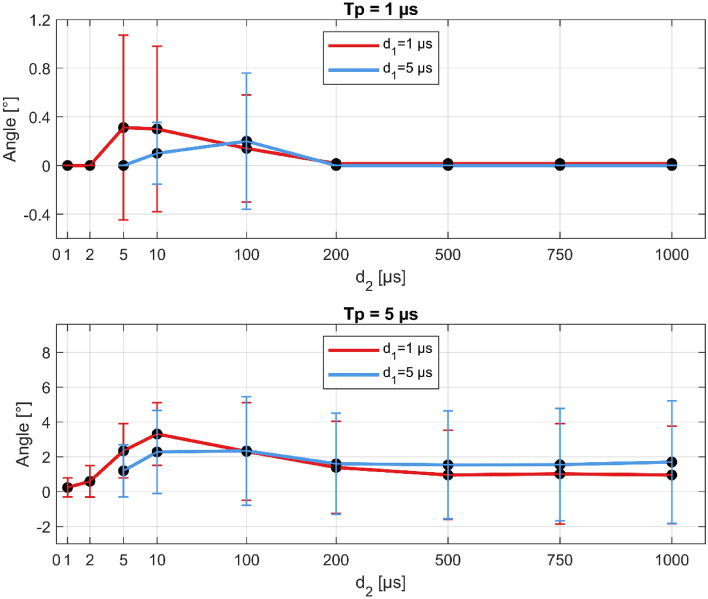
Longer interpulse delays reduce muscle contraction (response angle). Upper figure: 400 × 1 µs pulses, lower figure: 80 × 5 µs pulses. Note different ordinate scales (higher angles for T_p_ = 5 µs). The results are shown as the mean (black dots) ± standard error (vertical bars). T_p_-pulse width, d_1_-interphase delay, d_2_-interpulse delay.

Twelve additional pulse protocols were tested with extended interpulse delay, d_2_ = 200, 500, 750, and 1000 µs when the interphase delay (d_1_) with pulse width were set to either 1 or 5 µs. The muscle contraction responses are shown in Fig. 4 for pulse width (T_p_) of 1 µs (upper figure) and 5 µs (lower figure). The results show that as d_2_ increases from 1 µs to 10 µs for d_1_ = 1 µs, the angle of ankle dorsiflexion is increasing and reaching a peak (for d_1_ = 5 µs the peak is at 100 µs). Beyond 10 µs for d_1_ = 1 µs, and 100 µs for d_1_ = 5 µs, the angle of ankle dorsiflexion is decreasing, meaning that the threshold for muscle stimulation is higher for interpulse delays above 100 µs. For interpulse delays of 5 and 10 µs, the angle is the highest, meaning that the threshold for muscle stimulation is reduced. While for a pulse width of 1 µs the muscle contraction response reaches zero for d_2_ above 200 µs (upper figure), for pulse widths of 5 µs although the muscle contraction response is reduced, it does not completely disappear (lower figure). However, higher muscle contraction responses are observed only for the pulse protocols in the orange cluster (pulse protocols with a T_p_ of 5 µs and d_1_ and d_2_ up to 10 µs; see Table S1 in the Supplementary files). A difference can also be observed between d_1_ of 1 and 5 µs (red and blue lines). Interestingly, for a pulse width of 1 μs, there is slightly higher muscle contraction response for d_2_ of 5 μs and 10 μs. On the other hand, for a T_p_ of 5 μs, muscle stimulation with higher d_1_ (5 μs) first reduces the muscle contraction response (angle) and as d_2_ increases above 100 μs, the muscle contraction response is increasing, meaning that the muscle stimulation threshold is decreasing.

**Figure 5:**
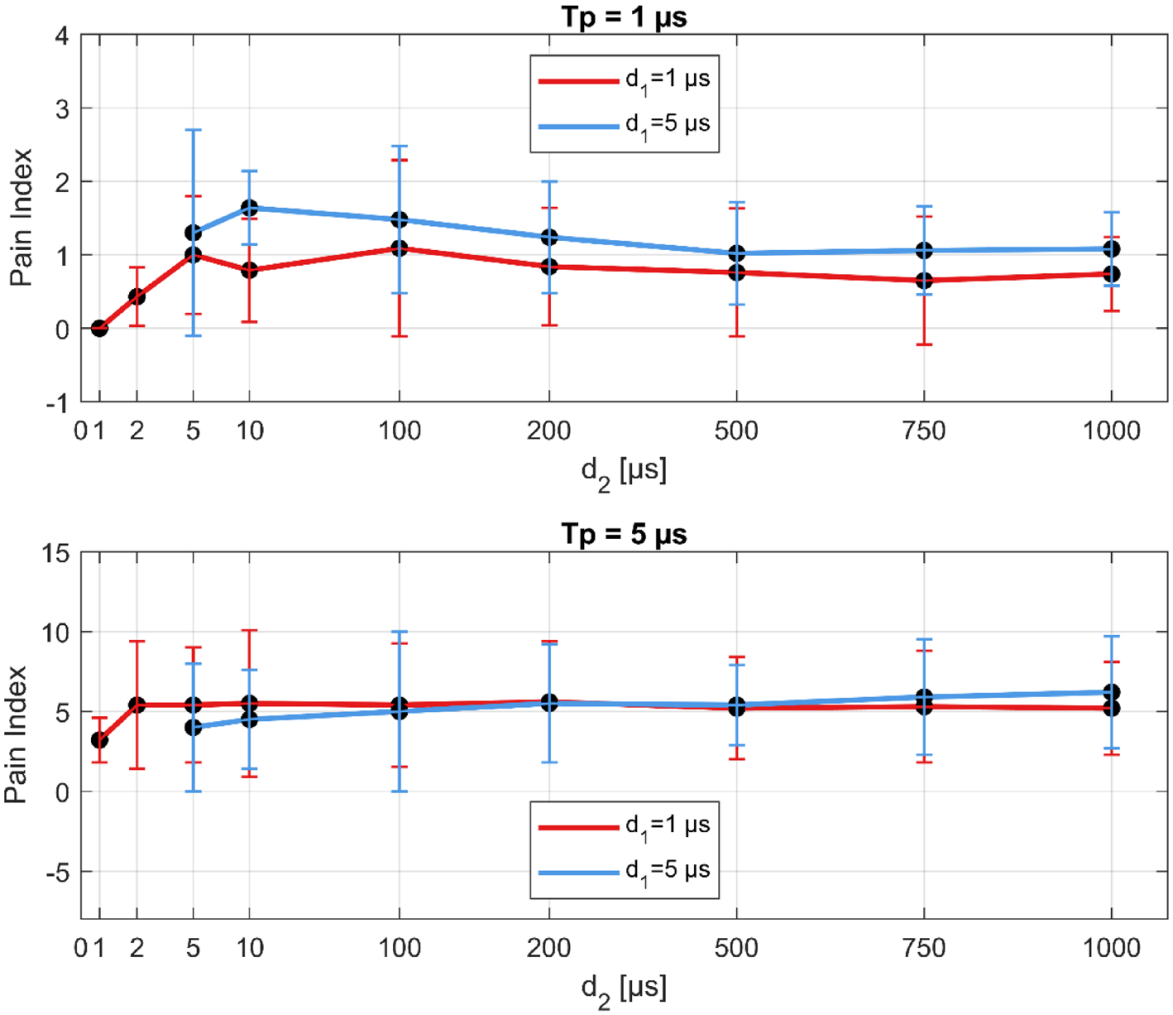
Longer interpulse delays slightly increase the pain index for longer pulse widths (lower figure). Upper figure: 400 × 1 µs pulses, lower figure: 80 × 5 µs pulses. Note different ordinate scales (higher pain indexes for T_p_ = 5 µs). The results are shown as the mean (black dots) ± standard error (vertical bars). T_p_-pulse width, d_1_-interphase delay, d_2_-interpulse delay.

The trends observed in Fig. 4 suggest (in agreement with a recent numerical study) that for reduced muscle contraction responses, shorter interphase delays with longer interpulse delays are preferred^72^. However, as shown on the lower graph in Fig. 5 and observed in the previous sub-sections, extending the interpulse delay beyond 10 µs for longer pulse widths results in higher pain indexes (as observed in Fig. 3, red asterisks).

### Interchanged interphase (d_1_) and interpulse delays (d_2_)

**Figure 6:**
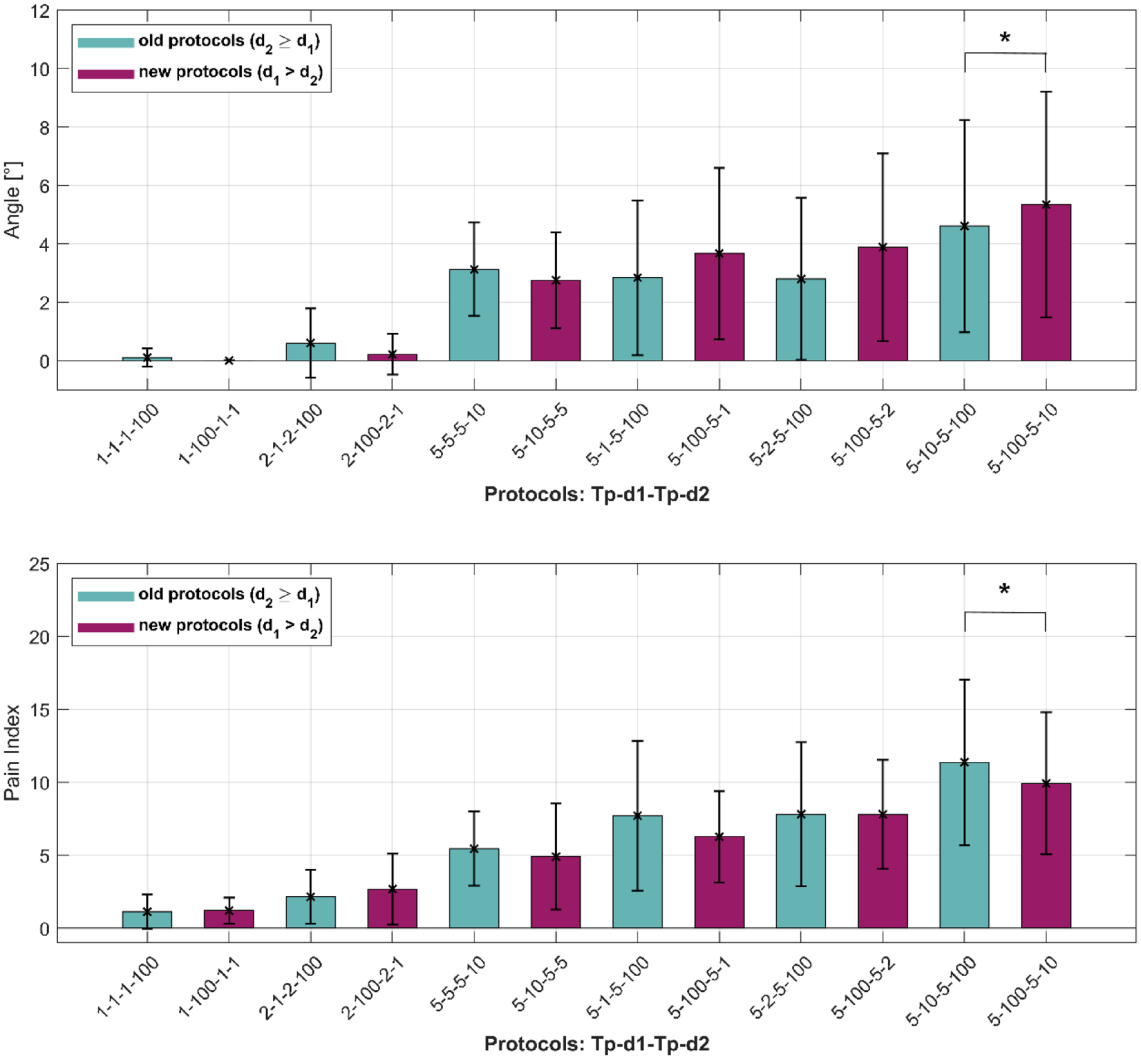
Interchanged interphase (d_1_) and interpulse delays (d_2_). Each bar represents one pulse protocol (T_p_-d_1_-T_p_-d_2_). Turquoise bars are already established biphasic pulse protocols (d_2_ ≥ d_1_), purple bars are the biphasic pulse protocols generated when d_1_ and d_2_ were interchanged (d_1_ > d_2_). The results are shown as the mean value (bar’s height) ± standard error (black vertical bars). The asterisks (*) show statistically significant differences between the pulse protocols (*P* < 0.05).

In Fig. 6, the interphase (d_1_) and interpulse delays (d_2_) are interchanged. Six biphasic pulse protocols out of the previously tested pulse protocols were chosen (old pulse protocols (d_2_ ≥ d_1_), turquoise bars in Fig. 6) for which d_1_ and d_2_ were interchanged (new pulse protocols (d_1_ > d_2_), purple bars in Fig. 6). Figure 6 shows the mean results with corresponding standard errors for muscle contraction response (upper figure) and pain index (lower figure). Paired t-test was performed (with a level of significance set to 0.05) within each set for both muscle contraction response and pain index. The results show that a statistically significant difference is observed between pulse protocols 5–10-5–100 and 5–100-5–10. Interchanging d_1_ from 10 to 100 and d_2_ from 100 to 10 induces higher muscle contraction responses but reduces the pain (pain index).

### Pain descriptors

**Figure 7:**
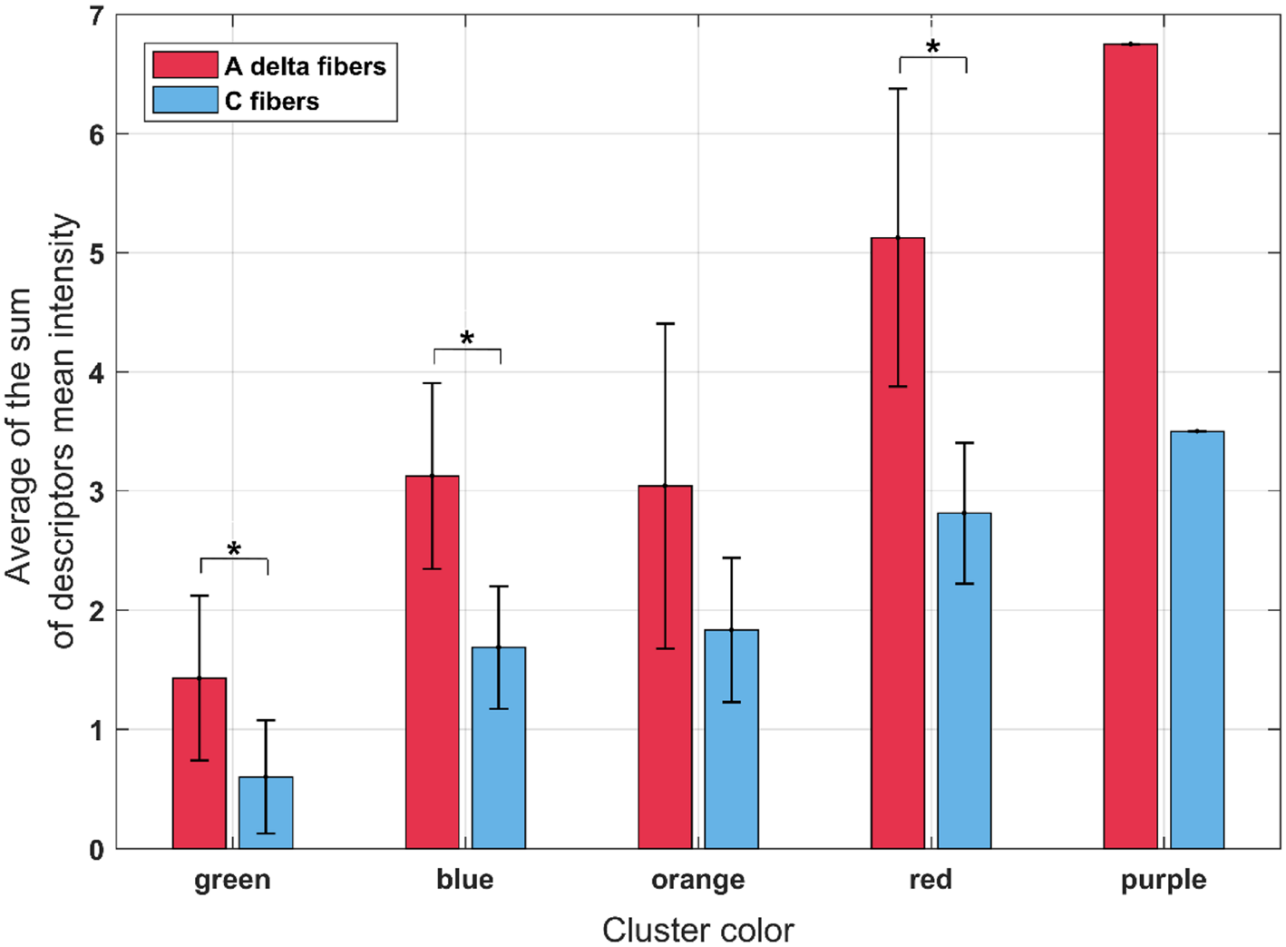
Sum of descriptors mean intensity for three chosen descriptors of both type of nerve fibers: A-delta (red bars) and C-fibers (blue bars). The data is shown as the average value (bar’s height) ± standard error (black vertical bars) for all biphasic pulse protocols included in a particular cluster. The asterisks (*) show statistically significant difference between the nerve fibers in the cluster (*P* < 0.05). Note that the purple cluster is only one pulse protocol cluster, thus the standard error is zero.

As described in the Methods section, three descriptors were chosen for each type of nerve fiber (A-delta and C-fibers). Descriptors mean intensity from each individual was calculated for each biphasic pulse protocol. For the chosen three descriptors for each type of nerve fiber, a sum of the descriptor mean intensity was calculated separately for A-delta and C-fibers. Depending on the generated clusters (Fig. 3), an average for the biphasic pulse protocols in the same cluster was calculated from the sum of the mean intensities for each pulse protocol. Figure 7 presents a bar graph for all five clusters showing the average values of the sum of descriptors’ mean intensity for each cluster with standard errors. The purple cluster (last two bars in Fig. 7) consists of a single pulse protocol, and hence the standard error is zero. Red bars show the average of the sum of descriptors mean intensity for A-delta fibers (descriptors: shooting, stabbing, and sharp), while the blue bars show the average of the sum of descriptors mean intensity for C-fibers (descriptors: throbbing, cramping, aching). A comparison between the average values of the nerve fibers within each cluster was performed using paired t-test (with a level of significance set to 0.05). The results show a statistically significant difference between the nerve fibers for the green, blue, and red clusters, indicating that more A-delta fibers are excited/stimulated by these pulse protocols.

## Discussion

This study represents the first study in humans examining both muscle contraction and pain sensation during high-frequency electroporation pulses. The aim of the study was to examine high-frequency, biphasic pulse protocols, which reduce muscle contraction responses in healthy individuals. High-frequency biphasic pulses in the range of microseconds with both symmetric and asymmetric interphase and interpulse delays were tested. These pulses were recently suggested to reduce the muscle contraction and pain sensation during electroporation-based therapies, in order to enable treatments without the need of muscle relaxants and anesthesia.

Our results obtained in healthy individuals confirm that very short, biphasic high-frequency pulses significantly reduce the muscle contraction response and pain sensation. Interphase delay (between the positive and negative phase) and interpulse delay (between the pulses) however, play a significant role in reducing the muscle contraction response and pain sensation. Very short interphase and interpulse delays (1 or 2 µs) reduce muscle contraction response and pain sensation even at the largest pulse width tested, i.e., 5 µs. Increasing both interphase and interpulse delays to 10 µs increases the muscle contraction response without strong pain sensation. However, in comparison to the amplitude determining protocol, i.e., reference protocol (8 monophasic pulses × 100 µs, 5 kHz with 2.5 times lower amplitude), these muscle contractions are still lower (Fig. 3).

Further increase of the interpulse delay (beyond 10 µs) additionally reduces the muscle contraction but increases the pain sensation (Fig. 4). This indicates that muscle contraction does not necessarily correlate to the pain sensation and vice versa. This may be due to different types of nerve fibers involved in the transmission of the signals-A-alpha motor fibers for muscle movement, and A-delta and C-fibers for transmitting nociception signals^58,59,73^.

Reduced muscle contractions have been achieved in multiple in vivo studies with application of biphasic pulses with pulse widths from 1 to 10 μs (of each phase) but only with equal interphase and interpulse delays with a duration of 1 to 5 μs^42,46,48,49,52,74^. Modifications of the interphase and interpulse delays have not been investigated as a method to reduce excitation within the H-FIRE protocols until recently when a theoretical argument for the extended interpulse delay while minimizing the interphase delay was presented^72^. Our results confirm that extending the interpulse delay while shortening the interphase delay indeed increases the muscle stimulation thresholds, meaning that the muscle contraction responses are reduced. However, the trends observed in our study indicate that extended interpulse delay, does not reduce the pain experienced by the individuals during the delivery of such pulses. On the contrary, longer interpulse delays were reported to be more painful (Figs. 4 and 5).

The pain estimation in the study was based on patient self-reporting using a clinically validated tool—the Short-Form McGill Pain Questionnaire (SF-MPQ). With this approach, we calculated the pain index and determined the pain descriptors for each pulse protocol. Based on the chosen pain descriptors the type of pain fibers that are predominantly excited was determined. Our results indicate that the A-delta nerve fibers are predominantly excited based on the chosen pain descriptors from the pain questionnaires. For each cluster, more A-delta fibers are excited/stimulated, suggesting that with short, biphasic high-frequency pulses there is higher A-delta nerve fibers involvement in transmitting nociception. However, for the orange cluster, no statistically significant difference occurred between the fibers (Fig. 7). The reason for this may be that these biphasic pulse protocols had higher muscle contraction responses. Thus, the individuals chose the “cramping” descriptor more often, which is a descriptor indicating C-fibers involvement. In the purple cluster there were more pain descriptors indicating A-delta involvement, however, this is a single pulse protocol only and no statistical analysis could be performed. Higher involvement of A-delta fibers can be due to the higher speed of pulse propagation in myelinated fibers, which also have a larger diameter than unmyelinated C-fibers. Moreover, C-fibers have longer chronaxie than the A-delta, indicating that the C-fibers require stronger stimulus (higher threshold amplitude) for excitation^58,59,73^. However, this may change if higher amplitudes would be used.

The location of the pain sensation for the short pulses with short interphase and interpulse delays was just a slight sensation right at the stimulation site, whereas for the longer interpulse delays, the individuals expressed the sensation as ‘’spreadable’’ along the muscle (leg) and longer lasting.

Slight redness at the site of the electrodes was visible immediately after the measurements, which disappeared within few hours. Namely, none of the individuals reported any visible signs of injury/redness at the site of the electrodes six hours after the treatment. More importantly, with the overall present pain intensity (PPI) index (scale: 0–5) being low (average: 0.7) the treatment was reported as tolerable and none of the individuals withdrew from the study.

Our study also shows that shorter interphase delays increase the stimulation threshold (Strength-Duration (S-D) curves, Fig. 2). The addition of a secondary anodic pulse to achieve balanced charge biphasic stimuli increases the threshold amplitude. This effect becomes greater as the interphase delay approaches 1 µs. However, for a biphasic pulse with longer interphase delays, i.e., 100 µs, the S-D curve is very close to the S-D curve for a single monophasic pulse, which is in agreement with existing literature^75–77^. As expected, for longer pulse widths, i.e., T_p_ = 50 µs, a single monophasic pulse resulted in a higher threshold amplitude than a single biphasic pulse for all interphase delays tested because a single biphasic pulse consists of two pulses (positive and negative), i.e., a monophasic pulse is 1xT_p_ long and a biphasic pulse is 2xT_p_ long.

Originally, all biphasic pulse protocols had interpulse delay longer or equal to the interphase delay. Additional measurements were therefore performed with interchanged delays to confirm that the approach d_2_ ≥ d_1_ is acceptable (Fig. 6). In the future, with this approach, the number of additional experiments may be reduced, as there were no statistically significant differences observed except for one of the tested sets of pulse protocols (5–10-5–100 and 5–100-5–10) where the muscle contraction response was higher for the interchanged delays (5–100-5–10), while the pain index was lower. Lower pain index at higher muscle contraction responses can be explained by the Gate Control Theory of Pain mechanisms. According to this theory, large fiber activity excites the inhibitory neurons, which diminishes the transmission of pain information. When there is more large fiber activity involved (A-alpha and A-beta fibers) in comparison to small fiber activity (A-delta and C-fibers), people tend to experience less pain^78–80^. This means that a non-painful input (e.g., a touch/massage on a bumped area) closes the nerve “gates” to the painful input because it increases the activity of the large fibers (A-beta fibers from the skin) and thus, prevents the pain sensation (lower activity of the pain fibers) from reaching the central nervous system. In our case, this would mean that stimulation of the muscle and the resulting muscle contraction activates/excites the large fibers and thus, reduces the excitation of the nociceptive (pain) fibers, i.e., the gates close. However, the theories and models of pain are still evolving and need further validation^81^.

### Limitations and drawbacks of the study

Firstly, although high-frequency electroporation protocols usually consist of more bursts of pulses delivered in succession, stimulation in our study was performed with only one burst of pulses. In addition, the total on-time of the pulses was always equal (800 µs). Second, the number of participants was limited to 25, which is enough for statistical analysis of trends, but not in-depth analysis between the pulse protocols. Moreover, the participants were in two different age groups and genders (younger-up to 32 years and elder-from 52 to 58 years; 12 male and 13 female), which also caused differences in the sensitivity. Namely, elder individuals tended to have slightly higher sensitivity (higher muscle contraction responses). This was also observed among the males compared to the females for the same biphasic pulse protocols. Therefore, relatively high standard errors and non-normal distribution of the results were observed. However, there was no statistically significant difference between the individuals’ responses (obtained in Design Expert v.12), which is in agreement with existing literature^82–84^. Third, the pain questionnaire (SF-MPQ) used, although already established in practice with validated Slovenian translation, some pain descriptors were hardly understandable to some individuals. Some of the pain descriptors were also non-applicable for this kind of study and were never chosen to describe the pain sensation. Hence, the choice of only three pain descriptors for each type of nerve pain fiber for assessing selectivity (A-delta and C-fibers). Last but not least, the voltage used for the biphasic pulse protocols was established based on the reference protocol (2.5 times higher than the amplitude for the reference protocol, since higher amplitudes are required to obtain comparable effect as with monophasic pulses^42,56,65^ at the same total on-time). The voltages used throughout the study were however low comparing to the voltages currently used for e.g., tissue ablations. Therefore, the VAS level (scale: 0–10) was lower (below 1) than in actual therapy^28,41,85,86^. However, we chose this approach to avoid potential damage to the underlying tissue, as we were testing 30 different biphasic pulse protocols per individual, which was almost an hour of repeated muscle stimulation. Therefore, performing the treatment with clinically relevant high voltage pulse protocols, on different tissue (tumors or heart) or locations (deep or superficial) remains to be established.

## Conclusion

In conclusion﻿, with our study we confirmed the hypothesis that using short (1 µs, 2 µs), biphasic high-frequency pulses with short interphase and interpulse delays reduces the muscle contraction in healthy individuals. We also demonstrated that these pulse protocols reduce the pain sensation. However, the interplay between the pulse width, interphase, and interpulse delays is more complex, and modification of these parameters results in either reduced muscle contraction response or pain sensation. Pain is not necessarily induced as a consequence of the muscle contraction response and vice versa. Namely, higher pain indexes are observed for pulse parameters that do not cause high muscle contraction response. Therefore, modification of the pulse parameters should be performed for a particular application of electroporation to reduce these effects, while providing safe, effective, and successful therapy.

## Supplementary Information

Supplementary Information.

## Data Availability

The data that support the findings of this study are available in the paper and its supplementary information files. The raw data are available from the authors upon reasonable request.
