# Supplementary material for: Muscle contractions and pain sensation accompanying high-frequency electroporation pulses
Source: Sci Rep. 2022 May 16;12:8019. doi: 10.1038/s41598-022-12112-9 (PMC9110404; doi:10.1038/s41598-022-12112-9)
Supplement: Supplementary file 1 — Supplementary Information. [file 41598_2022_12112_MOESM1_ESM.pdf]

Supplementary Information

Manuscript title: Muscle contractions and pain sensation accompanying high-frequency electroporation pulses
Authors: Aleksandra Cvetkoska, Alenka Maček-Lebar, Peter Trdina, Damijan Miklavčič and Matej Reberšek

- ***Table of biphasic pulse protocols***

***Table S1**. Table of tested biphasic pulse protocols with suitable coloring according to the cluster they belong to. P. No – protocol number, N – number of pulses, Tp – pulse width, d_1 – interphase delay, d_2 – interpulse delay*

P. No.	N	T_p	d_1 [µs]	d_2 [µs]
1	400	1	1	1
2	400	1	1	2
3	400	1	1	5
4	400	1	1	10
5	400	1	1	100
6	400	1	2	2
7	400	1	2	5
8	400	1	2	10
9	400	1	2	100
10	400	1	5	5
11	400	1	5	10
12	400	1	5	100
13	400	1	10	10
14	400	1	10	100
15	400	1	100	100
16	200	2	1	1
17	200	2	1	2
18	200	2	1	5
19	200	2	1	10
20	200	2	1	100
21	200	2	2	2
22	200	2	2	5
23	200	2	2	10
24	200	2	2	100
25	200	2	5	5
26	200	2	5	10
27	200	2	5	100
28	200	2	10	10
29	200	2	10	100
30	200	2	100	100

P. No.	N	T_p	d_1 [μs]	d_2 [μs]
31	133	3	1	1
32	133	3	5	5
33	133	3	5	800
34	100	4	1	1
35	100	4	5	5
36	100	4	5	800
37	80	5	1	1
38	80	5	1	2
39	80	5	1	5
40	80	5	1	10
41	80	5	1	100
42	80	5	2	2
43	80	5	2	5
44	80	5	2	10
45	80	5	2	100
46	80	5	5	5
47	80	5	5	10
48	80	5	5	100
49	80	5	10	10
50	80	5	10	100
51	80	5	100	100

- ***Hierarchical cluster tree (Dendrogram)***

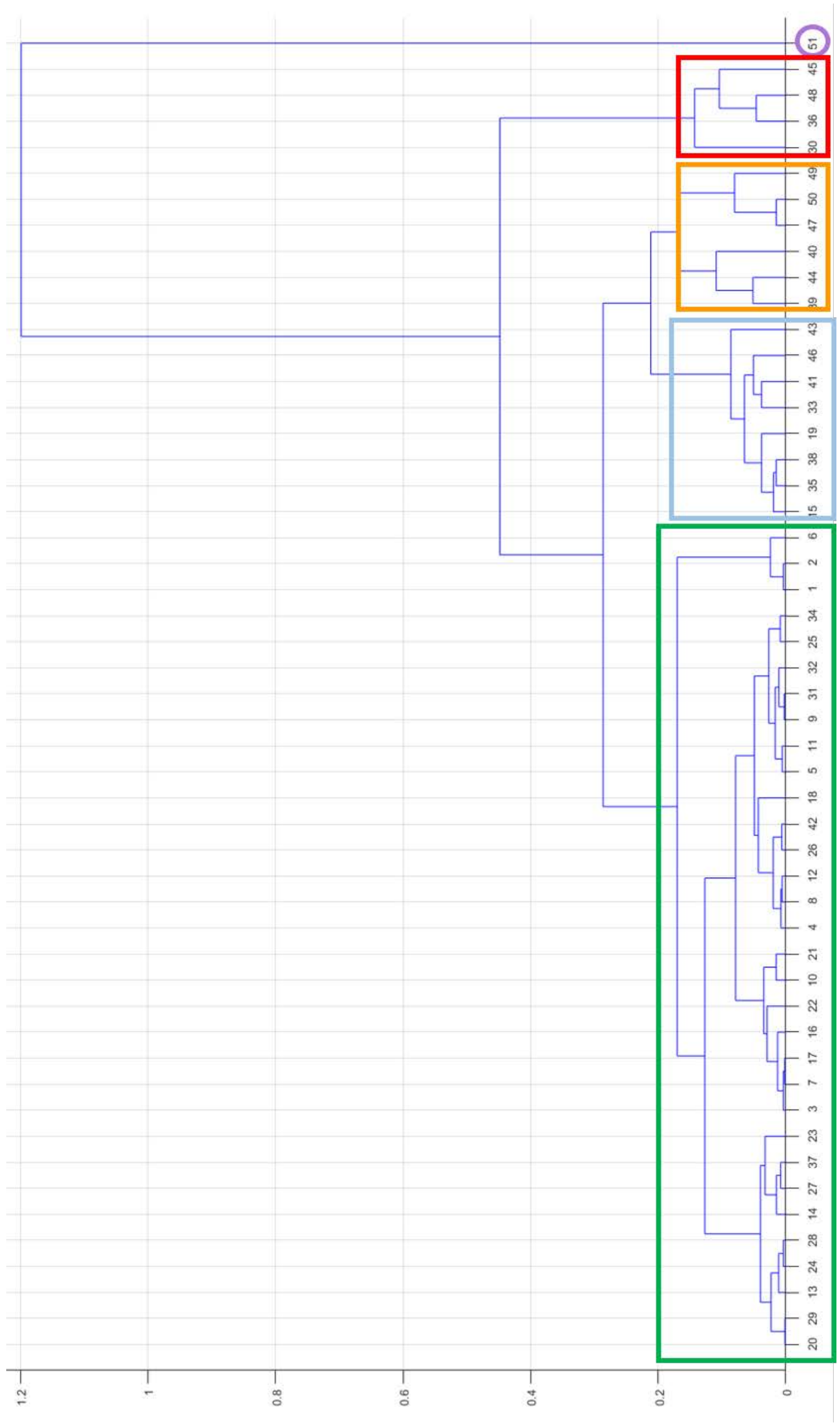

Figure S1. *Hierarchical cluster tree of the pulse protocols gathered in five clusters by color*

- **Multi-comparison analysis (N-way ANOVA)**

Multiple comparison test for three factors (pulse width, interphase, and interpulse delay) using the Dunn and Sidak's approach was performed to find the mean estimates and comparison intervals among the pulse protocols and thus, find statistically significant pulse protocols (separately for muscle contraction responses and pain indexes). Figures S2 and S4 show the performed multiple comparison test for muscle contraction responses and pain index, respectively. By selecting each pulse protocol separately (marked with blue line), all statistically significant pulse protocols were marked with red lines and the number of pulse protocols was shown below the graph.

Figure S2 provides an example for the pulse protocol 5-10-10 (T_p-d_1-d_2) marked with a blue line for which the mean estimates of the red marked pulse protocols are statistically lower. Note that the data is transformed with inverse square root transformation, thus the switched values on the graph (the red lines are shown with higher values). Figure S4 provides an example for the pulse protocol 5-100-100 (T_p-d_1-d_2) marked with a blue line for which the mean estimates of the red marked pulse protocols are statistically lower.

Figures S3 and S5 are the matrixes of the mean transformed muscle contraction responses and pain index, respectively (red numbers along the diagonal). The colored cells are marked accordingly to the pulse protocol where statistically significant difference occurs (derived from figures S2 and S4). An orange cell shows that the observed pulse protocol from the first column has a statistically higher mean value than the pulse protocol observed in the corresponding column of the cell (from the first row). A blue cell shows that the observed pulse protocol from the first row has a statistically lower mean value than the pulse protocol observed in the corresponding column of the cell (from the first row).

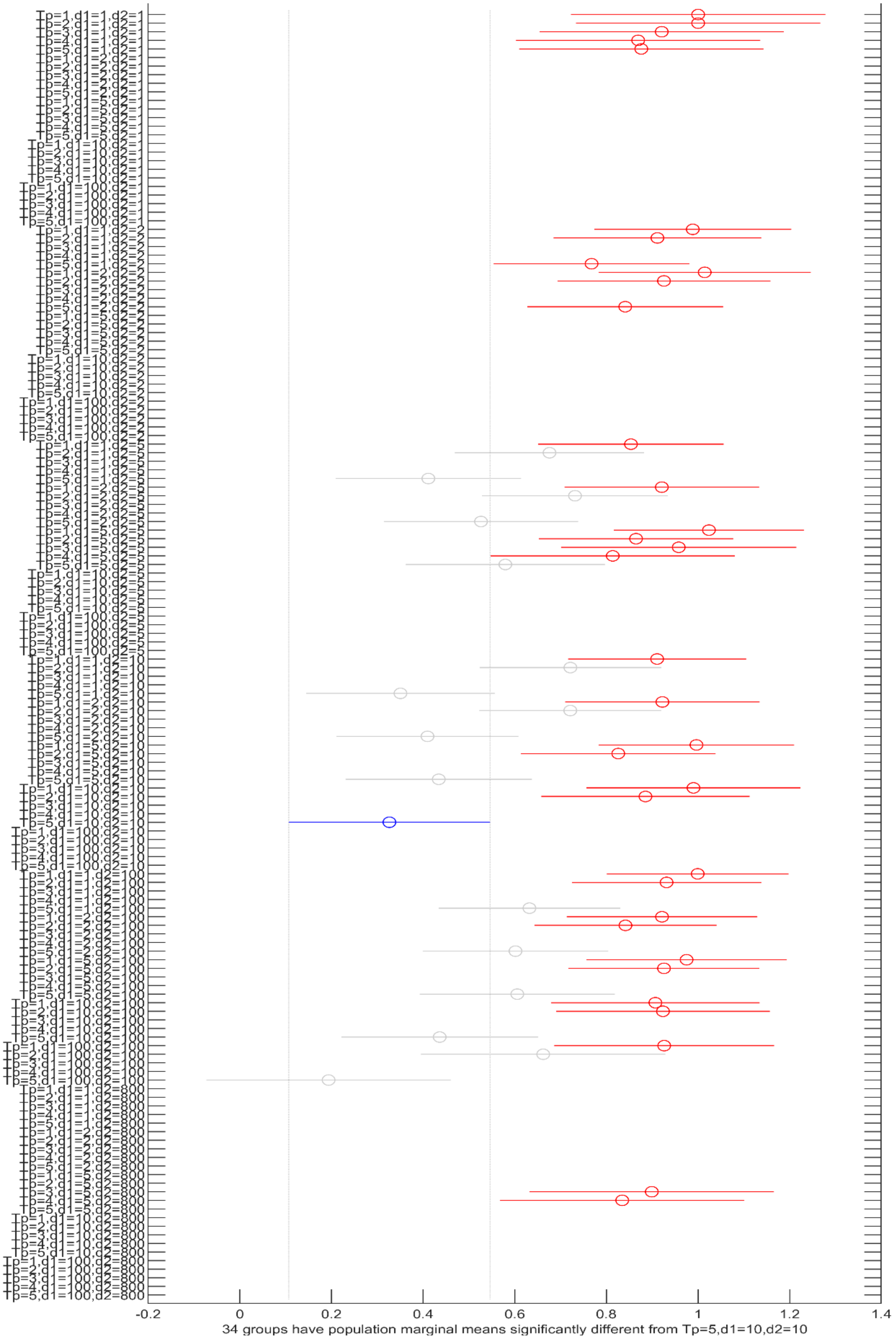

Figure S2. *Multiple comparison test for muscle contraction responses' data (inversely transformed) between the pulse protocols showing all statistically different pulse protocols (red lines) from the observed pulse protocol (marked with blue line).*

RESPONSES (int, 51), transform	Tp=1, D1=1, D2=1	Tp=2, D1=1, D2=1	Tp=3, D1=1, D2=1	Tp=4, D1=1, D2=1	Tp=5, D1=1, D2=1	Tp=1, D1=1, D2=2	Tp=2, D1=1, D2=2	Tp=5, D1=1, D2=2	Tp=1, D1=2, D2=2	Tp=2, D1=2, D2=2	Tp=5, D1=2, D2=2	Tp=1, D1=1, D2=5	Tp=2, D1=1, D2=5	Tp=5, D1=1, D2=5	Tp=1, D1=2, D2=5	Tp=2, D1=2, D2=5	Tp=5, D1=2, D2=5	Tp=1, D1=5, D2=5	Tp=2, D1=5, D2=5	Tp=3, D1=5, D2=5	Tp=4, D1=5, D2=5	Tp=5, D1=5, D2=5	Tp=1, D1=1, D2=10	Tp=2, D1=1, D2=10	Tp=5, D1=1, D2=10	Tp=1, D1=2, D2=10	Tp=2, D1=2, D2=10	Tp=5, D1=2, D2=10	Tp=1, D1=5, D2=10	Tp=2, D1=5, D2=10	Tp=5, D1=5, D2=10	Tp=1, D1=10, D2=10	Tp=2, D1=10, D2=10	Tp=5, D1=10, D2=10	Tp=1, D1=1, D2=100	Tp=2, D1=1, D2=100	Tp=5, D1=1, D2=100	Tp=1, D1=2, D2=100	Tp=2, D1=2, D2=100	Tp=5, D1=2, D2=100	Tp=1, D1=5, D2=100	Tp=2, D1=5, D2=100	Tp=5, D1=5, D2=100	Tp=1, D1=10, D2=100	Tp=2, D1=10, D2=100	Tp=5, D1=10, D2=100	Tp=1, D1=100, D2=100	Tp=2, D1=100, D2=100	Tp=5, D1=100, D2=100	Tp=3, D1=5, D2=800	Tp=4, D1=5, D2=800
Tp=1, D1=1, D2=1	1																																																		
Tp=2, D1=1, D2=1		1																																																	
Tp=3, D1=1, D2=1			0.9206																																																
Tp=4, D1=1, D2=1				0.8692																																															
Tp=5, D1=1, D2=1					0.876																																														
Tp=1, D1=1, D2=2						0.9883																																													
Tp=2, D1=1, D2=2							0.9112																																												
Tp=5, D1=1, D2=2								0.7675																																											
Tp=1, D1=2, D2=2									1.0144																																										
Tp=2, D1=2, D2=2										0.9254																																									
Tp=5, D1=2, D2=2											0.841																																								
Tp=1, D1=1, D2=5												0.8534																																							
Tp=2, D1=1, D2=5													0.6757																																						
Tp=5, D1=1, D2=5														0.4114																																					
Tp=1, D1=2, D2=5															0.9208																																				
Tp=2, D1=2, D2=5																0.7313																																			
Tp=5, D1=2, D2=5																	0.5262																																		
Tp=1, D1=5, D2=5																		1.0236																																	
Tp=2, D1=5, D2=5																			0.8645																																
Tp=3, D1=5, D2=5																				0.9577																															
Tp=4, D1=5, D2=5																					0.8137																														
Tp=5, D1=5, D2=5																						0.5793																													
Tp=1, D1=1, D2=10																							0.9105																												
Tp=2, D1=1, D2=10																								0.7213																											
Tp=5, D1=1, D2=10																									0.3505																										
Tp=1, D1=2, D2=10																										0.922																									
Tp=2, D1=2, D2=10																											0.721																								
Tp=5, D1=2, D2=10																												0.4095																							
Tp=1, D1=5, D2=10																													0.9964																						
Tp=2, D1=5, D2=10																														0.8257																					
Tp=5, D1=5, D2=10																															0.4341																				
Tp=1, D1=10, D2=10																																0.9899																			
Tp=2, D1=10, D2=10																																	0.8851																		
Tp=5, D1=10, D2=10																																		0.3264																	
Tp=1, D1=1, D2=100																																			0.9991																
Tp=2, D1=1, D2=100																																				0.9321															
Tp=5, D1=1, D2=100																																					0.6319														
Tp=1, D1=2, D2=100																																						0.9212													
Tp=2, D1=2, D2=100																																							0.8415												
Tp=5, D1=2, D2=100																																								0.6015											
Tp=1, D1=5, D2=100																																									0.9749										
Tp=2, D1=5, D2=100																																										0.9255									
Tp=5, D1=5, D2=100																																											0.6054								
Tp=1, D1=10, D2=100																																												0.9067							
Tp=2, D1=10, D2=100																																													0.9233						
Tp=5, D1=10, D2=100																																														0.4361					
Tp=1, D1=100, D2=100																																															0.926				
Tp=2, D1=100, D2=100																																																0.6619			
Tp=5, D1=100, D2=100																																																	0.1937		
Tp=3, D1=5, D2=800																																																		0.8989	
Tp=4, D1=5, D2=800																																																			0.8344

Figure S3. *Matrix of mean muscle contraction responses (transformed data) for each pulse protocol (red values along the diagonal) showing statistically different pulse protocols (orange cell – higher mean; blue cell – lower mean) as derived from figure S2.*

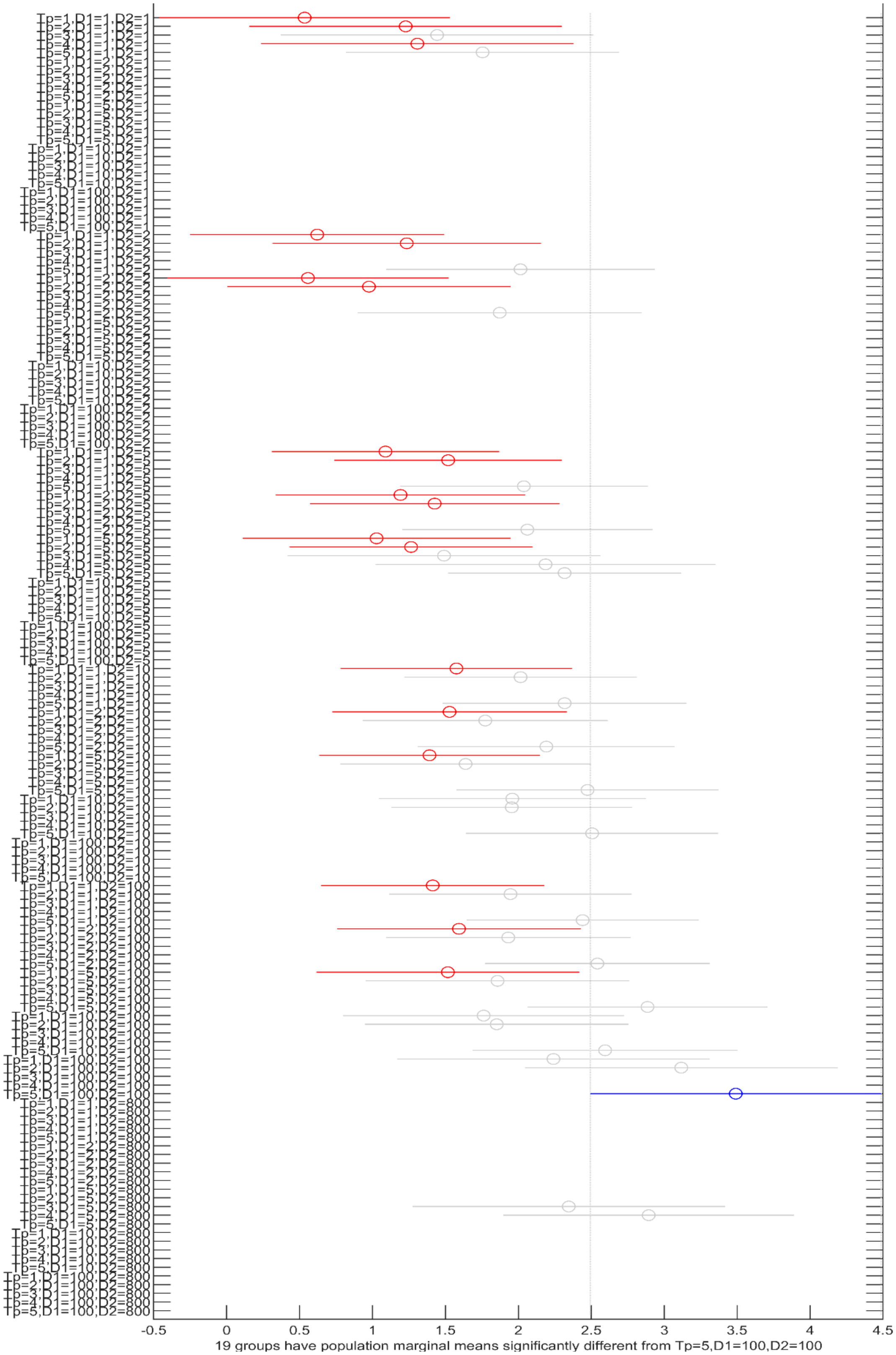

Figure S4. *Multiple comparison test for pain indexes' data (transformed) between the protocols showing all statistically different protocols (red lines) from the observed protocol (marked with blue line).*

PAIN INDEX (int, 51), transform	Tp=1, D1=1, D2=1	Tp=2, D1=1, D2=1	Tp=3, D1=1, D2=1	Tp=4, D1=1, D2=1	Tp=5, D1=1, D2=1	Tp=1, D1=1, D2=2	Tp=2, D1=1, D2=2	Tp=5, D1=1, D2=2	Tp=1, D1=2, D2=2	Tp=2, D1=2, D2=2	Tp=5, D1=2, D2=2	Tp=1, D1=1, D2=5	Tp=2, D1=1, D2=5	Tp=5, D1=1, D2=5	Tp=1, D1=2, D2=5	Tp=2, D1=2, D2=5	Tp=5, D1=2, D2=5	Tp=1, D1=5, D2=5	Tp=2, D1=5, D2=5	Tp=3, D1=5, D2=5	Tp=4, D1=5, D2=5	Tp=5, D1=5, D2=5	Tp=1, D1=1, D2=10	Tp=2, D1=1, D2=10	Tp=5, D1=1, D2=10	Tp=1, D1=2, D2=10	Tp=2, D1=2, D2=10	Tp=5, D1=2, D2=10	Tp=1, D1=5, D2=10	Tp=2, D1=5, D2=10	Tp=5, D1=5, D2=10	Tp=1, D1=10, D2=10	Tp=2, D1=10, D2=10	Tp=5, D1=10, D2=10	Tp=1, D1=1, D2=100	Tp=2, D1=1, D2=100	Tp=5, D1=1, D2=100	Tp=1, D1=2, D2=100	Tp=2, D1=2, D2=100	Tp=5, D1=2, D2=100	Tp=1, D1=5, D2=100	Tp=2, D1=5, D2=100	Tp=5, D1=5, D2=100	Tp=1, D1=10, D2=100	Tp=2, D1=10, D2=100	Tp=5, D1=10, D2=100	Tp=1, D1=100, D2=100	Tp=2, D1=100, D2=100	Tp=5, D1=100, D2=100	Tp=3, D1=5, D2=800	Tp=4, D1=5, D2=800
Tp=1, D1=1, D2=1	0.535																																																		
Tp=2, D1=1, D2=1		1.2276																																																	
Tp=3, D1=1, D2=1			1.4429																																																
Tp=4, D1=1, D2=1				1.3077																																															
Tp=5, D1=1, D2=1					1.7548																																														
Tp=1, D1=1, D2=2						0.621																																													
Tp=2, D1=1, D2=2							1.2349																																												
Tp=5, D1=1, D2=2								2.0147																																											
Tp=1, D1=2, D2=2									0.5575																																										
Tp=2, D1=2, D2=2										0.9759																																									
Tp=5, D1=2, D2=2											1.8722																																								
Tp=1, D1=1, D2=5												1.0892																																							
Tp=2, D1=1, D2=5													1.5189																																						
Tp=5, D1=1, D2=5														2.0377																																					
Tp=1, D1=2, D2=5															1.1921																																				
Tp=2, D1=2, D2=5																1.4264																																			
Tp=5, D1=2, D2=5																	2.0616																																		
Tp=1, D1=5, D2=5																		1.0279																																	
Tp=2, D1=5, D2=5																			1.2648																																
Tp=3, D1=5, D2=5																				1.4905																															
Tp=4, D1=5, D2=5																					2.1864																														
Tp=5, D1=5, D2=5																						2.317																													
Tp=1, D1=1, D2=10																							1.5753																												
Tp=2, D1=1, D2=10																								2.0153																											
Tp=5, D1=1, D2=10																									2.3175																										
Tp=1, D1=2, D2=10																										1.5285																									
Tp=2, D1=2, D2=10																											1.7731																								
Tp=5, D1=2, D2=10																												2.1918																							
Tp=1, D1=5, D2=10																													1.3911																						
Tp=2, D1=5, D2=10																														1.6383																					
Tp=5, D1=5, D2=10																															2.4741																				
Tp=1, D1=10, D2=10																																1.9585																			
Tp=2, D1=10, D2=10																																	1.9548																		
Tp=5, D1=10, D2=10																																		2.5056																	
Tp=1, D1=1, D2=100																																			1.413																
Tp=2, D1=1, D2=100																																				1.9462															
Tp=5, D1=1, D2=100																																					2.4406														
Tp=1, D1=2, D2=100																																						1.5929													
Tp=2, D1=2, D2=100																																							1.9308												
Tp=5, D1=2, D2=100																																								2.5417											
Tp=1, D1=5, D2=100																																									1.5169										
Tp=2, D1=5, D2=100																																										1.8573									
Tp=5, D1=5, D2=100																																											2.8852								
Tp=1, D1=10, D2=100																																												1.7611							
Tp=2, D1=10, D2=100																																													1.8518						
Tp=5, D1=10, D2=100																																														2.5947					
Tp=1, D1=100, D2=100																																															2.2406				
Tp=2, D1=100, D2=100																																																3.1168			
Tp=5, D1=100, D2=100																																																	3.4902		
Tp=3, D1=5, D2=800																																																		2.3453	
Tp=4, D1=5, D2=800																																																			2.8935

Figure S5. *Matrix of mean pain indexes (transformed data) for each pulse protocol (red values along the diagonal) showing statistically different pulse protocols (orange cell – higher mean; blue cell – lower mean) as derived from figure S4.*
